# Report of a rare case of colon cancer complicated by anomalies of intestinal rotation and fixation: a case report

**DOI:** 10.4076/1757-1626-2-6555

**Published:** 2009-09-14

**Authors:** Antonio Brillantino, Luigi Marano, Michele Schettino, Francesco Torelli, Giuseppe Izzo, Angelo Cosenza, Luigi Monaco, Raffaele Porfidia, GianMarco Reda, Felice Foresta, Natale Di Martino

**Affiliations:** 8^th^ Department of General and Gastrointestinal Surgery, School of Medicine Second University of NaplesP.zza Miraglia 3 80138 NaplesItaly

## Abstract

**Introduction:**

The Situs viscerum inversus associated with anomalies of intestinal rotation and fixation is an extremely rare condition. To the authors’ knowledge, this is the first report of colon cancer associated with intestinal malrotation and mesenterium ileocolicum commune.

**Case presentation:**

A 34-year-old man with a 2-month history of diarrhea associated with abdominal pain and weight loss underwent abdominal ultrasonography, colonscopy with biopsies and abdominal computed tomography scan with intravenous contrast. A right colonic neoplasm was diagnosed, observed only at surgery, as neither computed tomography or ultrasonography showed the intestinal malrotation. Particularly, the third and the fourth part of the duodenum descended vertically, without Treitz’s ligament in support to the duodeno-jejunal flexure. The small bowel and the colon were located in the right and left side of the abdominal cavity, respectively.

**Conclusion:**

The anomaly of situs viscerum inversus influenced the surgical strategy in this case because of the vascular and lymphatic anomalies. Lymphatic vessels were therefore marked with subserosal injection of patent blue in the proximity of the tumor. Subsequently, right colectomy was performed. Colectomy extended from the distal ileum to the descending colon, by ligature of the right colic artery and vein at the origin from the superior mesenteric vessels. Patent blue guided lymphadenectomy was also performed with curative intent. Finally, a mechanical ileo-colic anastomosis was carried out. After right colectomy and ileo-descending anastomosis, the Ladd’s procedure for intestinal malrotation was unnecessary. The authors believe that this strategy, despite the anatomical difficulties, represents an effective procedure for the radical surgical treatment of the right colon cancer associated with anomalies of intestinal rotation and fixation.

## Introduction

The right position of the organs, called situs viscerum solitus (SVS), can have some changes during the embryonic development, leading to an anatomical abnormality called situs viscerum inversus (SVI). The situs viscerum inversus totalis (SVIT) represents the most common condition, with an incidence varying from 1 in 4000 to 1 in 50000 live births [1,2], being characterized by a complete transposition of the viscera from their normal anatomic sites. The situs viscerum inversus partialis (SVIP) is less frequent and can involve only one organ or groups of viscera [3]. As regards the abdomen, all the organs or the only intestine may be in inverted positions. This condition may be associated with the “mesenterium ileocolicum commune” [4], an abnormality of intestinal fixation, characterized by a common mesentery attaching the terminal ileum, caecum, ascending and right half of the transverse colon.

In this report we describe a rare case of colon cancer associated with intestinal malrotation and mesenterium ileocolicum commune.

## Case presentation

A 34-years-old Caucasian male of Italian nationality was admitted to our department complaining for a 2-months history of diarrhea associated with abdominal pain, progressing from the upper to the lower abdomen, and weight loss. The patient did not report any other previous disease or surgery.

**Figure 1.**
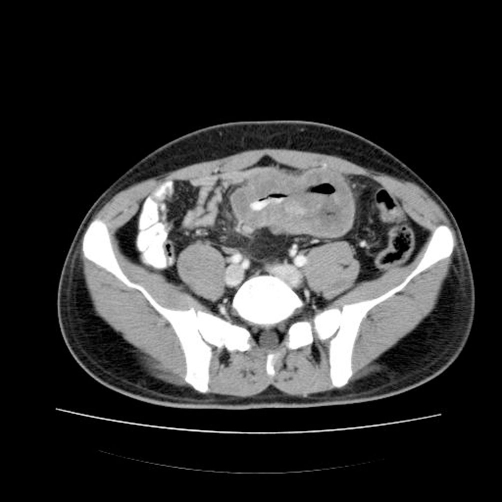
CT image showing some thickened ileal loops and an increased in volume mesenteric lymph node.

**Figure 2.**
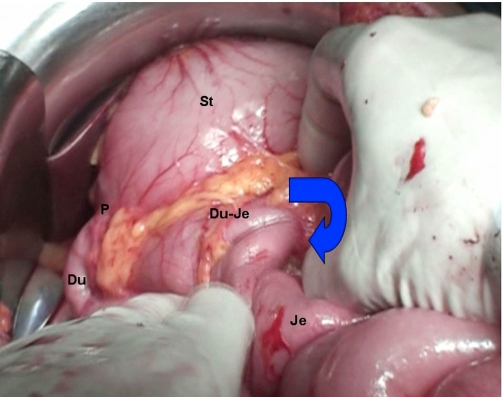
Intestinal malrotation; St: stomach, P: pylorus, D: duodenum, Je: jejunum.

**Figure 3.**
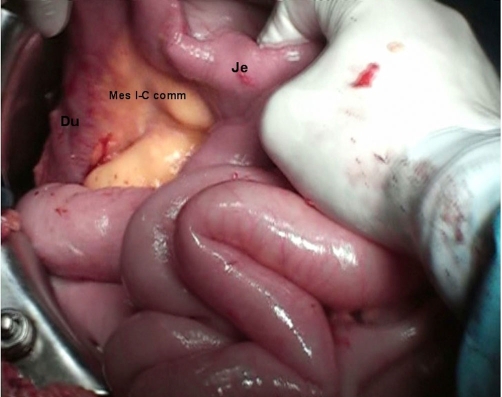
Mesenterium ileocolicum commune; Du: duodenum, Je: jejunum.

Moreover, no familiarity for metabolic or neoplastic diseases was found. The patient denied alcohol or tobacco use. Clinical examination revealed a distended abdomen, increased bowel sounds and a lightly painful mass at the left lower quadrant of the abdomen. Abdominal ultrasonography (US) showed a mass of 9 × 5 cm of mixed echogenicity in the proximity of the sigmoid colon with some hypoechoic lymph nodes of increased volume in the adipose adjacent tissue. Colonscopy with biopsy was performed because of the presumed diagnosis of intestinal neoplasm. Interestingly, at endoscopy, a polypoid vegetating mass, involving the 2/3 of the intestinal circumference was found, whereas, in the remaining colonic segments, no signs of mucosal injury were reported. At histopathologic standard examination, the mass showed microscopic aspects of adenocarcinoma. The abdominal computer tomography (CT) with intravenous contrast showed some thickened ileal loops and increased in volume mesenteric lymph nodes (Figure 1). At routine laboratory tests, no alterations of the principal biochemical parameters were found, except the levels of the following tumoral markers: CA 19-9 = 85.7 U/ml (normal value: up to 37 U/ml), CEA = 13.7 ng/ml (normal value: up to 3 ng/ml), CA 50 = 51.3 U/ml (normal value: up to 19 U/ml). Laparotomy was performed because of the diagnosis of malignant colonic neoplasm. At surgery a condition of intestinal malrotation was observed: the third and the fourth part of the duodenum descended vertically without Treitz’s ligament in support of the duodenojejunal junction and the small bowel and colon were located in the right and left side of the abdominal cavity, respectively (Figure 2). Particularly, the transverse colon did not clearly appear, being the ascending colon connected with the descending by a short segment of large intestine of 15 cm in length. The caecum and the right colon were attached through a long mesentery, which was a prolongation of the small bowel mesentery (Figure 3). In addition, the latter presented an anomalous curvilinear attachment, from the right side of the second lumbar vertebra to the left sacroiliac junction. For these reasons, although the neoplastic lesion was located in the caecum, it appeared placed in the left side of the abdomen, in the proximity of the sigmoid colon.

However, the supramesocolic organs maintained their normal position.

The presence of this anatomical abnormality strongly influenced some aspects of the surgical strategy. First of all, we decided, because of the associated vascular and lymphatic anomalies, to mark lymphatic vessels with peritumoral subserosal injection of patent blue violet, at the four cardinal points, to achieve a radical lymph node dissection. Therefore, right colectomy, extended from the distal ileum to the descending colon, by ligature of the right colic artery and vein at the origin from the superior mesenteric vessels, and patent blue guided lymphadenectomy, were performed, with curative intent. Subsequently, a mechanical ileo-colic anastomosis was carried out.

We did not perform the Ladd’s procedure [5] for the intestinal malrotation because, after an extended right colectomy and ileo-descending anastomosis, it was unnecessary.

Pathological examination revealed well-differentiated adenocarcinoma of the caecum infiltrating the subserosal layer without nodal involvement. The patient had an uneventful postoperative course.

## Discussion

Only several cases of colon cancer complicated by intestinal and vascular anomalies are reported in medical literature [6-8] and none of these was associated with mesenterium ileocolicum commune. This is the first report of a case of colon cancer associated with both intestinal malrotation and mesenterium ileocolicum commune.

Although the intestinal malrotation has long been recognized as a cause of intestinal obstruction in infants, it often remains asymptomatic in the adults and its real incidence is indeterminable [8]. Moreover, many cases of malrotation are incidentally revealed by laparotomy performed for coincidental diseases. Indeed, in our case, the intestinal malrotation was discovered only when laparotomy for the treatment of colon cancer was carried out.

Although the available literature suggests that the diagnosis of rotational anomalies can be made by means of barium enema [8], in the reported case, neither CT (performed without contrast in the small and large bowel) or US showed the intestinal malrotation. Furthermore, the contrast radiology was not carried out, because, after the endoscopic diagnosis of colonic neoplasm, it seemed unnecessary.

As regards the surgical technique, we preferred the laparotomy approach for two reasons. Firstly, nowadays, the laparoscopic approach is still not considered the gold standard for the treatment of the advanced colon cancer. Although the long-term outcome after laparoscopic and open surgery for colon cancer seems to be comparable, the choice of the best surgical approach is still source of doubts and the long-term oncological outcome, cost effectiveness and the impact on the patients’ quality of life should be defined by large-scale prospective randomized trials [9]. Secondly, the presence of not univocal radiologic and endoscopic findings led us to consider the laparotomy approach more safe and reliable.

At surgery, a radical and curative resection was obtained in spite of the anatomic anomalies and the altered vascular and lymphatic flow. Indeed, the extension of the lymphadenectomy was guided by the intraoperative injection of patent blue violet in the peritumoral subserosal layer, allowing an oncologically correct nodal dissection.

Moreover, the anomalous absence of a “true” transverse colon influenced the margins of resection that were extended from the distal ileum to the descending colon, including the short segment of large intestine that connected the ascending to the descending colon.

The surgical treatment of choice for the intestinal malrotation is the Ladd’s procedure. This includes the division of the Ladd’s band, the mobilization of the right colon and duodenum, the division of adhesions to the mesenteric base and appendectomy [5].

Obviously, the right colectomy performed for colon cancer and the presence of a floating duodenum, in the absence of a Treitz’s ligament attaching the duodeno-ingiunal junction, made the Ladd’s procedure unnecessary.

## Conclusion

The intestinal malrotation associated with mesenterium commune influenced the surgical strategy for the treatment of the associated colon cancer, because of the vascular and lymphatic anomalies. We believe that our case, despite the anatomical difficulties, illustrates an effective procedure for the oncologically correct surgical treatment of the colon cancer associated with anomalies of intestinal rotation and fixation.
